# Physiologically based pharmacokinetic model of two ALK inhibitors to inform drug and dosing regimens for brain metastasis patients

**DOI:** 10.3389/fphar.2026.1901617

**Published:** 2026-07-30

**Authors:** Na Yao, Jingpu Liu, Qian Xue, Jiankun Liu

**Affiliations:** 1 Department of Medical Oncology, Bethune International Peace Hospital, Shijiazhuang, China; 2 Department of Clinical Pharmacy, Bethune International Peace Hospital, Shijiazhuang, China; 3 Department of Medical Oncology, Heyou Hospital, Foshan, Guangdong, China

**Keywords:** ALK engagement ratios, brain metastases, brigatinib, lorlatinib, PBPK model

## Abstract

**Objective:**

This study developed and verified population Physiologically based pharmacokinetic (PBPK) models for brigatinib (BRI)and lorlatinib (LOR)to predict plasma and cerebrospinal fluid (CSF) pharmacokinetics (PK), assess brain penetration, and optimize dosing for brain metastases (BM).

**Methods:**

Population-based PBPK models were built by integrating *in vitro* and *in vivo* data, and verified against independent clinical datasets (plasma/CSF PK, ratio of unbound CSF to plasma concentrations [K_p,CSF_], and drug-drug interactions [DDIs]). Sensitivity analysis identified key parameters affecting CSF PK. An ALK engagement ratio (AER) ≥3.0 was applied to assess ALK mutant inhibition across different dosing regimens.

**Results:**

The PBPK models accurately predicted plasma concentration-time profiles and plasma/CSF PK parameters, with predicted-to-observed ratios for AUC_0-τ,ss_, C_max,SS_, and C_min,SS_ within the 0.5- to 2.0-fold range. Predicted K_p,CSF_ and CYP3A4/CYP2C8-mediated DDI outcomes also closely matched clinical data, confirming model reliability. For standard-dose BRI, AER values remained below 3.0, with the exception of that against C1156Y and I1171T. In specific ALK mutations (EML4-ALK, C1156Y, G1269A, and I1171T), mean AER values exceeded the efficacy threshold of 3.0 (red dotted line) under dosing regimens of 240 mg QD or 180 mg BID. For LOR, under the three dosing regimens (50 mg QD, 50 mg BID, and 100 mg QD), the simulated mean AER values against eight mutations exceeded the threshold of 3.0, with the exception of L1196M, G1202R, and I1171N at 50 mg QD.

**Conclusion:**

These verified PBPK models provide a mechanistic framework for individualized dosing optimization against specific ALK mutations and support using CSF concentrations as a surrogate for brain exposure.

## 1 Introduction

Lung cancer remains the leading cause of cancer-related mortality worldwide, imposing a disproportionate burden on global public health systems (Leiter et al., 2023). Non-small cell lung cancer (NSCLC) represents the most prevalent histological subtype, constituting approximately 85%–90% of all lung cancer diagnoses (Xia et al., 2024). The anaplastic lymphoma kinase (ALK) fusion oncogene has been identified as a critical therapeutic target (Schneider et al., 2023). ALK rearrangements are detected in approximately 3%–7% of NSCLC patients (Facchinetti et al., 2024), defining a distinct molecular subset characterized by a relatively younger age at onset.

Soda et al. first cloned and identified the EML4-ALK fusion gene in NSCLC patient samples, establishing its foundational role as a lung cancer driver mutation (Soda et al., 2007). Although the EML4-ALK fusion is the primary pathogenic alteration at diagnosis, the clinical application of first- and second-generation ALK tyrosine kinase inhibitors (TKIs)-such as crizotinib, ceritinib, and alectinib-inevitably leads to acquired resistance. Previous studies have systematically elucidated these resistance mechanisms. Initially, secondary point mutations within the ALK kinase domain (e.g., L1196M, C1156Y, and G1269A) were identified as direct causes of clinical failure following crizotinib treatment (Choi et al., 2010; Katayama et al., 2012). Subsequently, specific mutations including G1202R, I1171T, I1171S, and I1171N were frequently detected in tumors that developed resistance to second-line agents like alectinib (Recondo et al., 2020). Consequently, the most commonly documented secondary point mutations encompass C1156Y, L1196M, G1269A, G1202R, and the I1171 subvariants (T, S, and N).

A critical clinical complication in ALK-positive NSCLC is the high propensity for tumor cells to metastasize to the central nervous system (CNS). Brain metastases (BM) occur in approximately 24%–29% of ALK-positive NSCLC patients at initial diagnosis, and this proportion escalates to over 50% during the disease trajectory (Petrelli et al., 2018; Nelson and Wang, 2023). The CNS serves as a pharmacological sanctuary site due to the presence of the blood-brain barrier (BBB), making the management of BM a paramount challenge in ALK-positive NSCLC. To overcome this, next-generation ALK inhibitors, notably Brigatinib (BRI) and Lorlatinib (LOR), have been developed. BRI is a potent, selective ALK/ROS1 inhibitor designed to overcome multiple ALK resistance mutations (Markham, 2017), while LOR, a third-generation macrocyclic ALK inhibitor (Syed, 2019), was explicitly engineered for broad-spectrum activity against ALK resistance mutations and enhanced CNS penetration. Preliminary clinical studies have shown that both agents have demonstrated significant intracranial efficacy for ALK-positive NSCLC patients, particularly those with BM (Camidge et al., 2018; Garcia et al., 2024).

Adequate drug penetration into brain specifically, traversing the intact BBB to achieve therapeutic concentrations in the cerebrospinal fluid (CSF)-is a prerequisite for sustained *in vivo* target inhibition and the effective treatment of brain metastases (Nelson and Wang, 2023; Arvanitis et al., 2020). Selecting an ALK inhibitor with favorable CNS pharmacokinetics (PK) and designing an optimal dosing regimen will maximize the success rate of these novel agents in treating brain cancer. However, similar to many other novel or existing anticancer drugs, the CNS PK of these two ALK inhibitors in cancer patients remain critically underexplored and poorly understood. The current understanding of the spatiotemporal distribution and exposure of drugs within the CNS and brain tumors is largely inadequate. This knowledge gap primarily stems from the inherent challenges of clinical sampling and the limitations of currently available imaging and analytical technologies (Tregub et al., 2025); direct measurement of the spatiotemporal penetration and exposure of drugs in the human brain and brain tumors is practically unfeasible (Tregub et al., 2025). The lack of quantitative understanding of the systemic, CNS, and CSF spatial PK of systemically administered drugs renders the development of new therapeutics and the rational application of existing drugs for brain cancer a challenging, and often unsuccessful. Consequently, there is a need to develop a more reliable, mechanism-based approach to predict drug distribution within the human CSF.

Physiologically based pharmacokinetic (PBPK) modeling offers a robust solution to this challenge. PBPK modeling is a mechanistic approach that integrates human physiological parameters (e.g., organ volumes, blood flows, and the abundances of enzymes and transporters) with drug-specific parameters (e.g., physicochemical properties, *in vitro* metabolism, and transporter kinetics). This integration enables the prediction of PK behaviors at both systemic and organ levels (Khambhawala et al., 2025). Several studies have been published using PBPK models to predict drug concentrations in the human CSF (Li et al., 2023; Chen et al., 2024; Zhan et al., 2025). In this study, we developed and verified a whole-body PBPK model that incorporates the general anatomical structure of the human brain and the principal physiological processes governing drug distribution into the CSF. This mechanism-based model allows for the prediction of both plasma and CSF PK of ALK inhibitors following various dosing regimens. Our research provides quantitative insights to inform mutation-specific selection of ALK inhibitors and optimize corresponding dosing strategies, thereby facilitating the rational development and optimal clinical application of ALK inhibitors in the treatment of BM.

## 2 Methods

### 2.1 PBPK model structure and development

**FIGURE 1:**
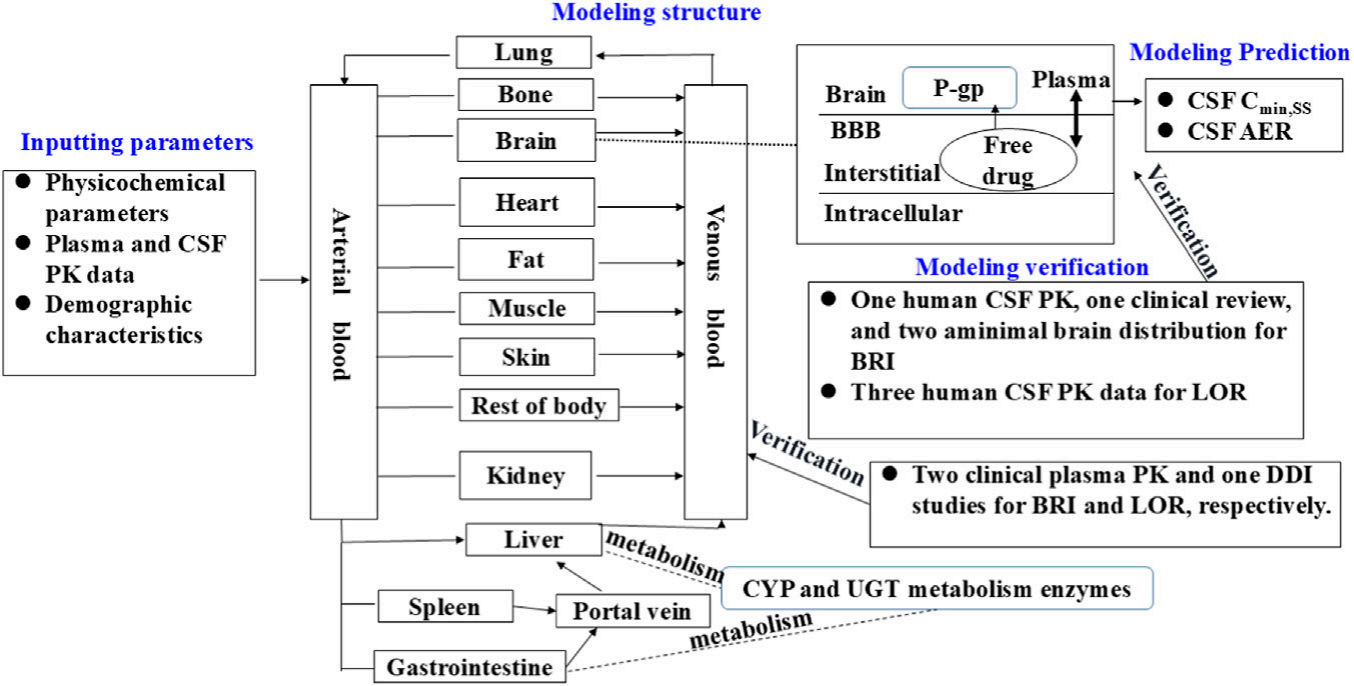
The schematic representation of the PBPK model. The PBPK framework integrates the gastrointestinal tract, a comprehensive circulatory system (encompassing arterial and venous pathways), eliminating organs (e.g., liver and kidneys), and non-eliminating tissues (e.g., lung and bone), all interconnected by blood flow. Metabolism of BRI and LOR occurs primarily in the gut and liver via CYP and UGT enzymes, while efflux transport at the blood-brain barrier is mediated by P-glycoprotein. The brain is represented as a distinct, multi-compartmental structure comprising tissue, plasma, blood cells, interstitial fluid, and intracellular space. Notably, the free concentrations within the interstitial fluid of these two ALK inhibitors were employed as a surrogate marker for free CSF levels. Model verification was achieved through the comparison of simulated results with clinical human plasma and CSF pharmacokinetic data, DDI studies, and animal brain distribution data.

To predict the plasma and CSF PK of BRI and LOR, a whole-body PBPK model was constructed in PK-Sim, incorporating the default four-compartment brain module (Version 12.1; Bayer Technology Services, Leverkusen, Germany). The physiological architecture of this framework connects the gastrointestinal tract, arterial and venous blood, eliminating organs (liver and kidneys), and non-eliminating tissues via a blood flow network. Tissue distribution was characterized using a permeability-limited approach, reflecting that tissue penetration is predominantly restricted by membrane permeability rather than blood flow. The default brain module was segmented into plasma, blood cell, interstitial, and intracellular spaces. Although the CSF and brain interstitial fluid are not physiologically identical compartments, consistent with prior PBPK methodologies (Li et al., 2023; Diestelhorst et al., 2013), interstitial fluid concentrations were utilized as a surrogate for CSF levels, thereby enabling the estimation of unbound inhibitor concentrations within the CSF. The PBPK framework is presented in Figure 1.

### 2.2 PBPK model parameters

The PBPK model was parameterized using a dataset comprising physicochemical properties, PK parameters, CYP3A4/P-gp interaction constants, and physiological parameters in cancer patients. The physiological and drug-specific parameters for BRI and LOR implemented in the PBPK model were primarily extracted from the literature (Li et al., 2023; Chen et al., 2024; Zhan et al., 2025; Li W. L. et al., 2018; PMDA, 2026; Ali et al., 2024; Hanley et al., 2024; Gupta et al., 2021; Li W. et al., 2018; Alsmadi et al., 2021), and these input values is provided in Supplementary Table S1. Gastrointestinal absorption of BRI and LOR was characterized using the built-in Weibull function in PK-Sim. To align the predicted absorption rate and extent with clinical observations by Gettinger et al. at 90 mg OD (Gettinger et al., 2016) and by Shaw et al. at 100 mg OD (Shaw et al., 2017), the Weibull absorption time was adjusted to 45 min for BRI and 30 min for LOR. Partition coefficients were calculated using the standard Rodgers and Rowland method, while cellular permeabilities were estimated via the PK-Sim method. Renal clearance (CL_R_) was calculated as the product of glomerular filtration rate (GFR) and unbound plasma fraction (f_up_). Furthermore, the P-gp transport parameter was calibrated to 0.1 nmol/min/g tissue from literature values (2.02 pmol/min) (Li W. et al., 2018) to recapitulate the observed clinical CSF concentrations of BRI. All remaining metabolic constants, along with enzyme and transporter expression levels, were extracted from published literature (Li et al., 2023; Zhan et al., 2025; Alsmadi et al., 2021).

Several studies have confirmed the differences in physiological parameters between healthy populations and cancer patients (Li et al., 2023; Zhan et al., 2025; Alsmadi et al., 2021). Specific disease-state adjustments were implemented in the PBPK model: (1) hepatic enzyme downregulation: relative to healthy subjects, hepatic CYP3A4 and CYP2C19 expression was reduced by 45% and 30%, respectively (Yang et al., 2023). This corresponded to absolute tissue concentrations of 3.02 μM/L for CYP3A4 and 0.51 μM/L for CYP2C19 (Alsmadi et al., 2021) (Supplementary Table S1). Although CYP2C8 is the primary metabolic enzyme for BRI *in vitro* metabolism experiment, literature reports show that its activity or expression levels in cancer patients are minimal or show no significant difference compared to healthy individuals (Yang et al., 2023). Therefore, no adjustment for CYP2C8 expression was made in this study. (2) altered blood biochemistry: pathological decreases in plasma albumin (from 4.5 to 3.1 g/dL) and hematocrit (from 0.43 to 0.33) were applied (Alsmadi et al., 2021). Consequently, cancer-specific free fraction in plasma (f_up_) and blood-to-plasma concentration ratio (R_bp_) values were recalculated using the established scaling equations (Alsmadi et al., 2021). (3) delayed gastric emptying: A prolonged mean gastric emptying time (GET) of 190 min was incorporated to reflect the delayed gastrointestinal motility reported in cancer patients (Alsmadi et al., 2021). Conversely, P-gp expression was maintained at baseline healthy levels, given the lack of significant disease-associated differences reported in the literature (Schwenger et al., 2018). Similarly, due to insufficient clinical evidence, the expression profiles of other uncharacterized metabolic enzymes were left unadjusted.

### 2.3 Clinically observed PK data in plasma and CSF

The plasma and CSF PK of two ALK inhibitors (BRI and LOR) were evaluated in clinical trials involving ALK-positive NSCLC patients (Gettinger et al., 2016; Shaw et al., 2017; Hanley et al., 2023; Sato et al., 2024; Tugnait et al., 2020; Chen et al., 2021; Sun et al., 2022; de Leeuw et al., 2023; Patel et al., 2020; Chen et al., 2020). Demographic data and specific dosing schedules are summarized in Table 1. All participants received repeated administrations until steady-state conditions were achieved. Specifically, PK data on BRI include plasma PK across various dosing levels from two clinical trials (Gettinger et al., 2016; Hanley et al., 2023), alongside CSF PK at a standard monotherapy dose from one study (Sato et al., 2024). Additionally, one trial assessed plasma PK when BRI was combined with CYP3A4 modulators and CYP2C8 inhibitors (Tugnait et al., 2020). A comparable pattern was observed for LOR, with two clinical trials evaluating plasma PK across multiple regimens (Shaw et al., 2017; Chen et al., 2021). Furthermore, three studies examined its CSF penetration at the standard monotherapy dose (Shaw et al., 2017; Sun et al., 2022; de Leeuw et al., 2023), while two others investigated plasma PK in the presence of CYP3A4 modulators (Patel et al., 2020; Chen et al., 2020).

**TABLE 1:** Studies included in PBPK model development and validation.

Drug	Clinical studies	PK	Study design	Demographic characteristics	Comments
BRI	Gettinger et al. (2016)	Plasma	Ascending different repeated-dosing regimens of 30, 60, 90, 120, 180, and 240 mg QD	A total of 100 patients were enrolled for PK assessment. Females accounted for 49%. The median age was 54 years (range: 44–64)	The clinical PK data from Gettinger et al. at 90 mg OD were used to optimize the PBPK model parameters. Additionally, the P-gp transport parameters were calibrated using data from Sato et al. The model was then validated using clinical PK data from Gettinger et al. at other dosing regimens, alongside other independent PK studies and DDI data
	Hanley et al. (2023)	Plasma	Repeated-dosing regimens of 180 mg QD	A total of 24 patients were enrolled in the study. The population was 54% female, with a median age of 57 years (range: 26–75 years) and a median weight of 67.5 kg (range: 44.4–129.0 kg)	
	Sato et al. (2024)	CSF	Starting at 90 mg daily for 7 days, then increased to 180 mg	A 34-year-old male patient was assessed	
	Tugnait et al. (2020)	DDI studies	Gemfibrozil 600 mg BID for 5 days, itraconazole 200 mg BID for 5 days, or rifampin 600 mg QD for 7 days and a single oral dose of BRI on day 5 of the CYP inhibitor/inducer	60 Subjects were randomized to receive a single oral dose of BRI alone in treatment period 1. In treatment period 2, subjects received multiple doses for DDIs. Subjects aged 18–65 years with a BMI of 18–33 kg/m2 and a minimum weight of 50 kg	
LOR	Shaw et al. (2017)	Plasma/CSF	Doses ranged from 10 mg to 200 mg QD. Plasma PK were evaluated at steady-state after repeated doses, and concentrations in CSF were measured on Day 8	The study patients consisted of 54 patients, with a gender distribution of 32 (59%) males and 22 (41%) females, a mean age of 52 ± 13years	The clinical PK data from Shaw et al. at 100 mg OD were used to optimize the PBPK model parameters to accurately simulate plasma and CSF concentrations. The model was then validated using clinical PK data from Shaw et al. at other dosing regimens, alongside other PK study by Chen et al., two CSF PK studies, and two DDI studies
	Chen et al. (2021)	Plasma	Escalating doses of 100 mg QD for patients	This study enrolled a total of 52 patients, with a female proportion of 58.4%. The age range was 18–83 years, with an average age of 53 years. The average height was 166 cm and the average weight was 68 kg, resulting in an average BMI of 24.7 kg/m^2^	
	Sun et al. (2022)	CSF	Patients receiving 100 mg QD	A total of 5 patients consisting of 3 females (60%) and 2 males, with ages ranging from 41 to 75 years. Their height ranged from 147.0 cm to 170.2 cm, weight ranged from 46.6 kg to 155.5 kg, and BMI ranged from 18.8 to 65.8 kg/m^2^	
	de Leeuw et al. (2023)	CSF	100 mg QD	A total of 7 patients, two receiving LOR (100 mg QD). The patients’ ages ranged from 49 to 70 years, with a female (50%)	
	Patel et al. (2020)	DDI studies	Participants received ITR 200 mg orally QD for 11 days, with a single dose of LOR administered on day 5 at 100 QD	16 male participants with a mean age of 34.1 years (range 20–54), body weight 74.6 kg (53.2–97.0), BMI 24.5 kg/m^2^ (19.5–30.4)	
	Chen et al. (2020)	DDI studies	All participants were to receive RIF 600 mg QD on days 1–12 and a single dose of LOR 100 mg QD on day 8	A total of 12 participants (11 men and 1 woman) aged 36.5 (21–55) years, with a body mass index of 25.4 (21.6–29.4) kg/m^2^ and a body weight of 76.2 (64.1–91.3) kg	

QD: once daily; BID: twice daily.

### 2.4 PBPK model validation

#### 2.4.1 Model verification based on PK in plasma and CSF compartments

The initial validation of the PBPK model’s predictive capability involved a comparison between simulated and real-world PK profiles after repeated dosing of two ALK inhibitors in patients (Gettinger et al., 2016; Chen et al., 2021). To quantify model accuracy, prediction-to-observation ratios were derived for steady-state PK parameters, specifically the area under the curve (AUC_0-τ,ss_), maximum concentration (C_max,ss_), and trough concentration (C_min,ss_) at steady-state. Successful model performance was defined as these ratios falling within 0.5- to 2.0-fold for prediction-to-observation ratios in plasma and in CSF. Additionally, as the ratio of unbound CSF to plasma concentrations (K_p,CSF_) is widely used to quantify brain penetration, the model-predicted K_p,CSF_ in the CSF compartments was compared with observed values in cancer patients.

#### 2.4.2 Model verification based on drug-drug interaction predictions

Based on the *in vitro* metabolism data, CYP2C8 and CYP3A4 were identified as the primary enzymes involved in BRI metabolism, with CYP2C8 being the predominant pathway. However, clinical drug-drug interaction (DDI) studies (Tugnait et al., 2020) indicated that CYP3A4 is the major metabolic enzyme for BRI *in vivo*. Therefore, the adjusted clearance data for CYP3A4 and CYP2C8 were obtained based on literature data (Hanley et al., 2024). Following this adjustment, CYP3A4 and CYP2C8 account for 79.1% and 20.9% of the total *in vivo* clearance from BRI converting to metabolite M36, respectively (Supplementary Table S2). LOR was predominantly metabolized by CYP3A4, CYP3A5, and UGT1A4, which convert to M6, M2a, and M1a, accounting for 100%, 55.6%, and 89.5% of the contributions, respectively. To further verify the pivotal roles of main enzymes in total clearance for BRI and LOR, a series of DDI simulations were performed. Initially, the plasma PK of BRI were simulated under co-administration with gemfibrozil (GEM, a strong CYP2C8 inhibitor), itraconazole (ITR, a strong CYP3A4 inhibitor), and rifampicin (RIF, a potent CYP3A4 inducer). Subsequently, the plasma PK of LOR was simulated alongside ITR and RIF. All PBPK model parameters for GEM, ITR, and RIF, as well as the CYP2C8 and CYP3A4 inhibition and induction parameters, were extracted from published literature (Ren et al., 2025; Türk et al., 2019). Given the absence of *in vitro* RIF induction EC_50_ and E_max_ data for UGT1A4, based on mRNA fold-change data, a 4.93-fold increase in hepatic UGT1A4-mediated metabolism was applied to model (Gufford et al., 2018). The dosing regimens applied in these DDI scenarios for the ALK inhibitors and their corresponding modulators were consistent with previously reported clinical studies (Tugnait et al., 2020; Patel et al., 2020; Chen et al., 2020) (Table 1).

Various dosing regimens outlined in Table 1 were applied in the simulations. To ensure consistency with the referenced clinical trials (Gettinger et al., 2016; Shaw et al., 2017; Hanley et al., 2023; Sato et al., 2024; Tugnait et al., 2020; Chen et al., 2021; Sun et al., 2022; de Leeuw et al., 2023; Patel et al., 2020; Chen et al., 2020), all modeling was conducted under fasting conditions using matched demographic parameters. In instances where specific patient characteristics were unreported, default values within the PK-Sim software were utilized as substitutes.

#### 2.4.3 Context of use and fit-for-purpose statement

The PBPK model developed in this study was verified for a specific context of use. Specifically, the model is fit for the purpose of qualitatively predicting the CSF distribution of BRI and LOR, and informing ALK mutation-specific dose adjustments in adult NSCLC patients with BM. The model’s predictive performance was verified against the clinical plasma and CSF PK data. However, this model has not been verified for use in pediatric populations, patients with severe renal or hepatic impairment. Furthermore, prospective clinical trials are warranted to fully verify the dosing recommendations generated from these simulations.

### 2.5 Sensitivity analysis of modelling parameters

A sensitivity analysis was conducted to determine how model parameters influenced key pharmacokinetic metrics, specifically steady-state CSF AUC0_-τ,ss_, C_max,SS_, and C_min,SS_. The procedure involved three steps: (1) selecting a predefined set of physiological and drug-dependent variables, including LogP, f_up_, Weibull time, enzyme-specific clearances (mediated by CYP3A4, CYP2C8, CYP3A5, CYP2C19, UGT1A3, and UGT1A4), P-gp efflux, P-gp K_i_, CYP3A4 E_max_, and albumin concentrations; (2) fluctuating each variable within a ±20% range; and (3) calculating the sensitivity coefficient (SC) for each input using a standard equation (Saeheng et al., 2020). Parameters yielding an absolute SC exceeding 1.0 were classified as having a substantial impact on the model.

### 2.6 Simulated ALK engagement ratios in CSF for two ALK inhibitors

Currently, there are no studies reporting the effective free concentration of BRI in CSF. However, studies have reported the effective concentration thresholds of LOR in CSF for different ALK mutations: the threshold concentration for wild - type ALK is 2.58 ng/mL, for L1196M is 21.08 ng/mL, and for G1202R is 67.06 ng/mL (Sun et al., 2022). Based on the methods reported in the literature (Li et al., 2021), the ALK engagement ratio (AER) was used as a crude predictor of efficacy, which is calculated as the unbound CSF concentration divided by the IC_50_ value. The effective concentrations of LOR in CSF for wild - type, L1196M, and G1202R correspond to AERs of 3.2, 1.5, and 3.3, respectively. Therefore, we set AER ≥ 3.0 as a predictor of efficacy with a high probability of clinical efficacy for BRI and LOR in patients with BM. IC_50_values were obtained from the published paper (Supplementary Table S3) (Sabari et al., 2017).

## 3 Results

### 3.1 Model validation for prediction of plasma PK

**FIGURE 2:**
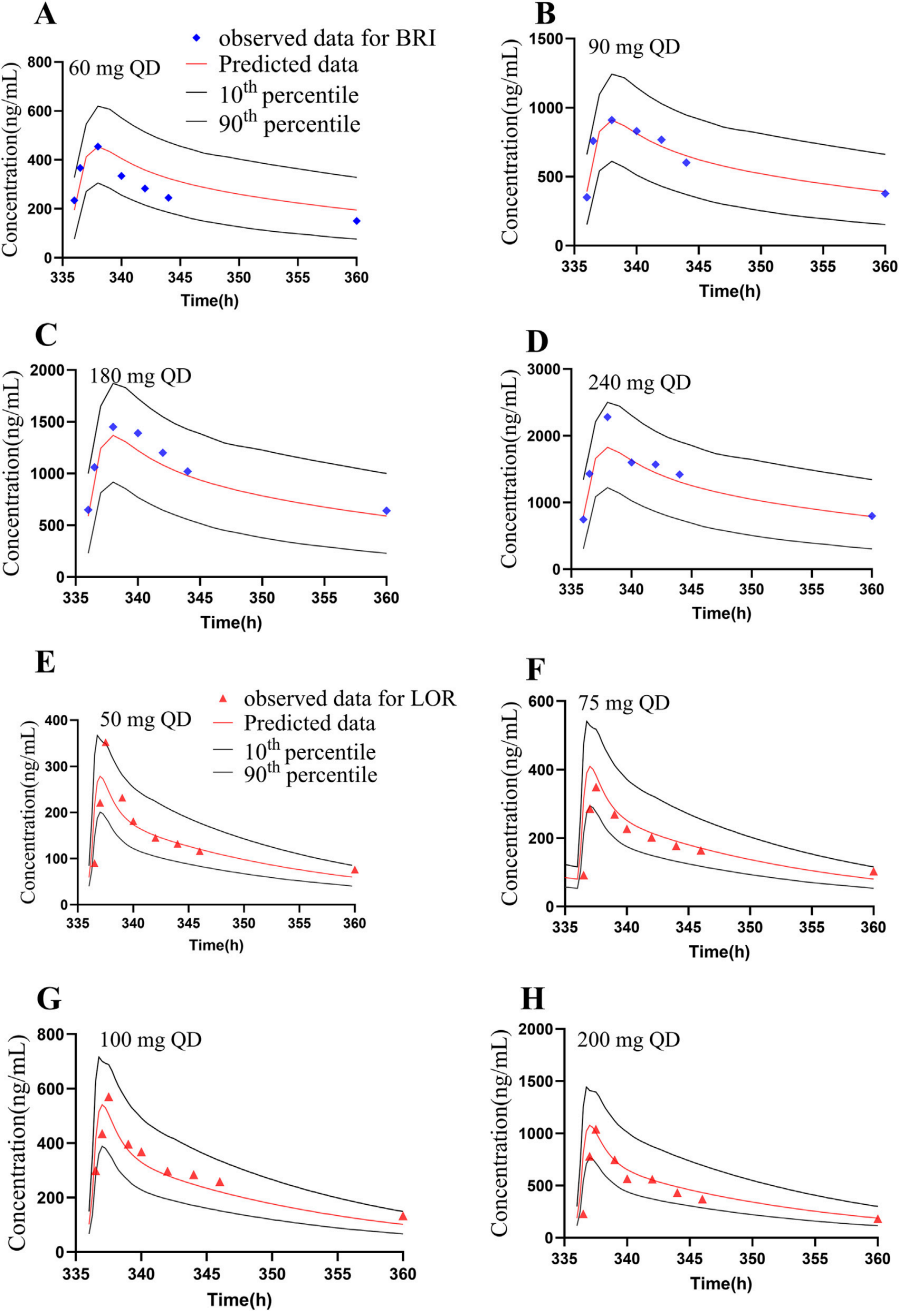
Predicted and observed plasma concentration-time profiles after 14 days of dosing. **(A-D)** BRI at 60, 90, 180, and 240 mg QD; **(E-H)** LOR at 50, 75, 100, and 200 mg QD. Solid blue squares represent the arithmetic mean observed plasma PK data for BRI, and solid red up-triangles represent the geometric mean observed plasma PK data for LOR. Solid red lines depict the predicted PK profiles (arithmetic mean for BRI; geometric mean for LOR), while solid black lines indicate the 10th and 90th percentiles of the predicted distributions across all dosing regimens. The period of 335–360 h on the x-axis represents the steady-state window.

**FIGURE 3:**
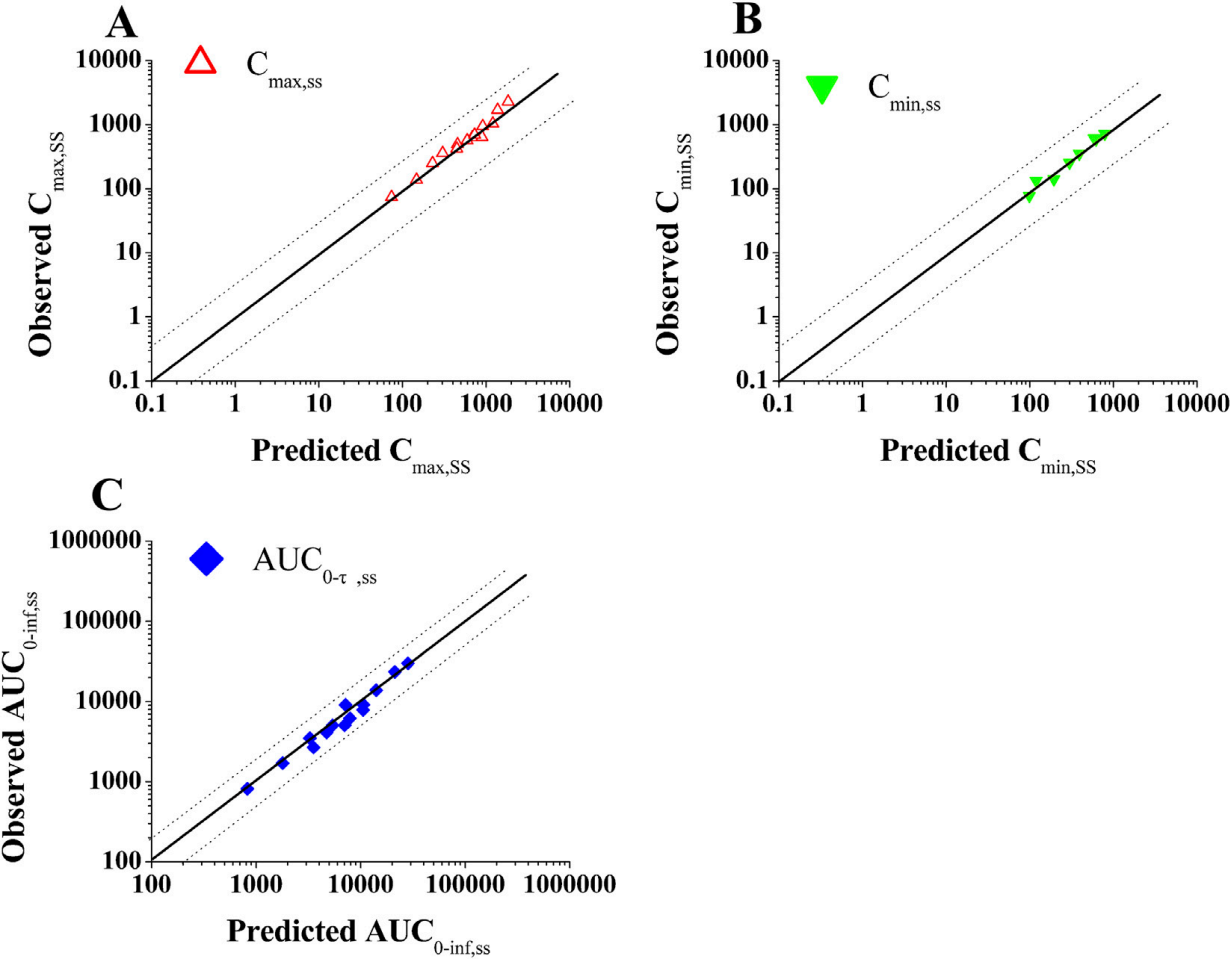
Goodness-of-fit plots for the BRI and LOR PBPK model, showing predicted versus observed **(A)** $C_{\max,\text{ss}}$ , **(B)** $C_{\min,\text{ss}}$ , and **(C)** $\text{AU}C_{0-\tau ,\text{ss}}$ . The line of identity and acceptable limits (0.5- to 2.0-fold) are depicted as solid and dashed lines, respectively. All predicted and observed plasma PK data for BRI and LOR were included in the analysis.

The PBPK-simulated plasma profiles for BRI and LOR are illustrated in Figure 2. The model accurately captured the plasma concentration-time profiles following repeated dosing, aligning well with published clinical data (Gettinger et al., 2016; Chen et al., 2021). To further verify the developed population PBPK models, the simulated results were compared against the four independent clinical dataset (Gettinger et al., 2016; Shaw et al., 2017; Hanley et al., 2023; Chen et al., 2021). As shown in Figure 3 and Supplementary Table S4, all predicted-to-observed ratios for the evaluated PK parameters fell within the 0.5- to 2.0-fold range. Notably, 89% of the ratios were within the 0.7–1.3 range, with a maximum ratio of 1.4 observed for C_max,SS_, demonstrating the model’s predictive accuracy.

### 3.2 Model validation for prediction of CSF PK

**FIGURE 4:**
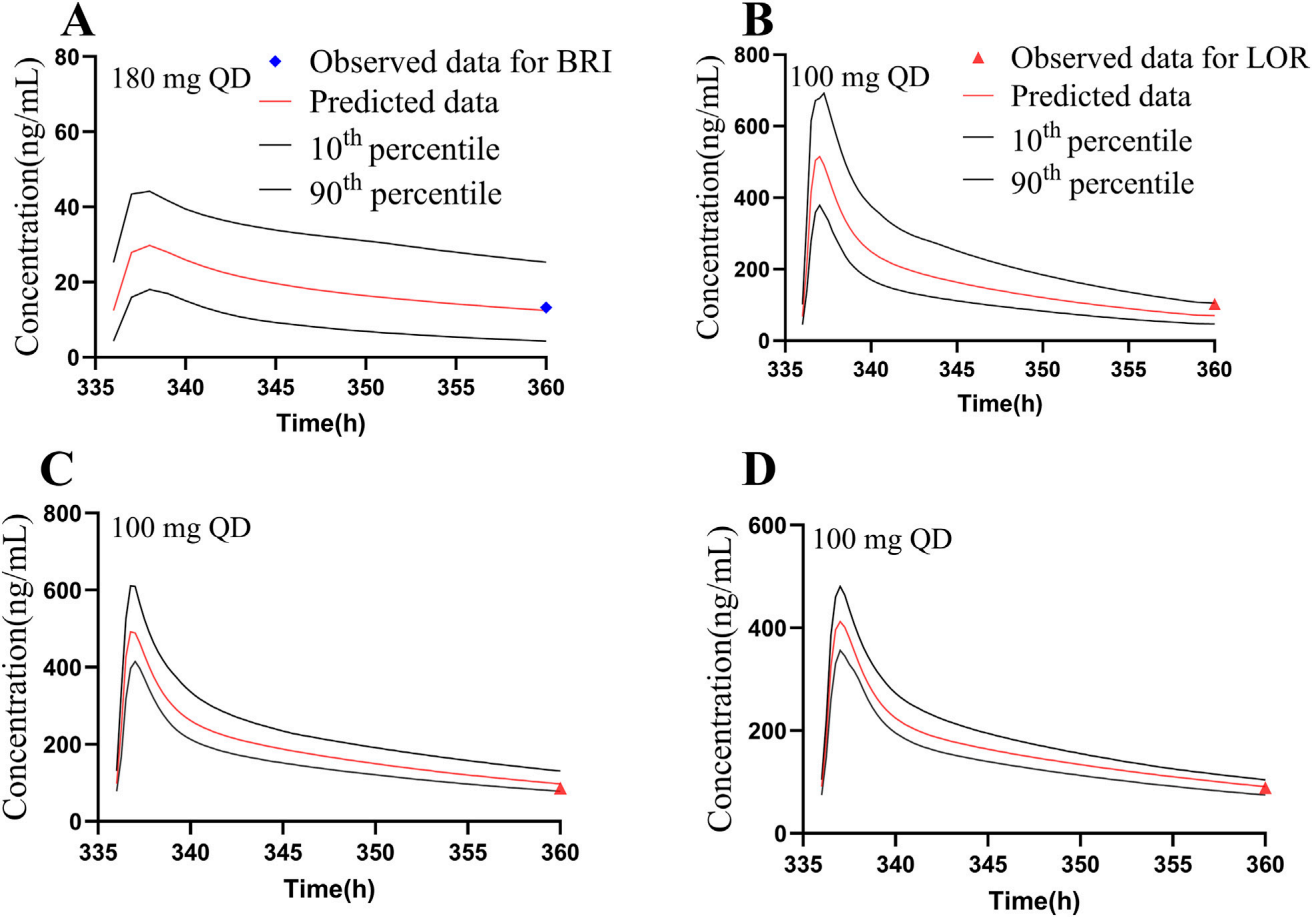
Predicted CSF concentration-time profiles after 14 days of dosing. **(A)** BRI at 180 QD; **(B–D)** LOR at 100 mg QD. Solid blue squares represent the arithmetic mean observed CSF C_min,ss_ for BRI, and solid red up-triangles represent the geometric mean observed CSF C_min,ss_ for LOR. Solid red lines depict the predicted PK CSF profiles (arithmetic mean for BRI; geometric mean for LOR), while solid black lines indicate the 10th and 90th percentiles of the predicted CSF PK profiles. The period of 335–360 h on the x-axis represents the steady-state window.

Figure 4 illustrates the PBPK-simulated CSF concentration-time profiles for BRI and LOR. The model successfully recapitulated the steady-state C_min,ss_ for both inhibitors following multiple dosing regimens. As detailed in Table 2, the predicted C_min,ss_ values demonstrated good agreement with the observed clinical data, yielding predicted-to-observed ratios within the acceptable 0.5- to 2.0-fold range (Shaw et al., 2017; Sato et al., 2024; Sun et al., 2022; de Leeuw et al., 2023). Specifically, the ratio for BRI was 0.93 (model calibration), while those for LOR across the three clinical studies were 0.91, 1.13, and 1.03, respectively. Furthermore, the accurate simulation of clinical K_p,CSF_ values further demonstrate the predictive capability of the developed model. Specifically, the predicted-to-observed K_p,CSF_ ratios were 0.20 (model calibration) vs. 0.13 for BRI. For LOR, the predicted and observed values from the three clinical studies were 0.63 vs. 0.77, 0.84 vs. 0.82, and 0.71 vs. 0.61. (Table 2). Due to the limited clinial data for BRI in CSF of human, the model was further verified using BRI distribution data from rat and mouse brain tissues (Supplementary Table S5). The results also show that the predicted brain-to-plasma exposure ratios are consistent with the experimental values (Sparidans et al., 2018; Li et al., 2019).

**TABLE 2:** Comparison of predicted and observed mean trough concentrations in CSF.

Clinical studies	Inhibitors	Dosing regimens	C_min,SS_ (ng/mL, CV %)		Prediction/observation ratio	K_p,CSF_	
			Prediction	Observation		Prediction	Observation
Sato et al.	BRI	180 mg QD	12.4 (8.1)	13.2	0.93	0.20	0.13^a^
Shaw et al.	LOR	100 mg QD	93.3 (61.9)	102.7 (21.5)	0.91	0.63	0.77
Sun et al.		100 mg QD	97.5 (26.2)	86.5 (36.8)	1.13	0.84	0.82
Leeuw et al.		100 mg QD	91.6 (13.4)	88.6 (38.7)	1.03	0.71	0.61

CV %: percentage coefficient of variation; C_ss,min_: trough steady-state concentration; K_p,CS_: mean CSF-to-plasma ratio; ^a^Calculated using the product of total plasma concentration and f_up_.

### 3.3 Model validation based on DDI predictions

The predicted DDI ratios derived from the PBPK models are detailed in Supplementary Table S6. All simulated AUC and C_max_ ratios exhibited good agreement with clinical observations (Tugnait et al., 2020; Patel et al., 2020; Chen et al., 2020). These successful DDI predictions confirm the reliability of the incorporated CYP3A4 and CYP2C8 metabolic parameters, thereby comprehensively validating the overall accuracy of the PBPK model.

### 3.4 Sensitivity analysis

**FIGURE 5:**
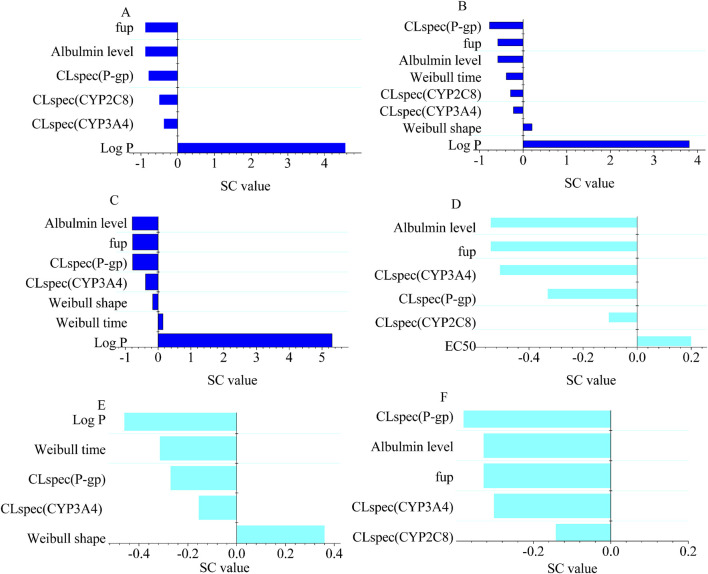
Sensitivity analysis of the PBPK models. Figure **(A–C)** for AUC_0-τ,ss_, C_max_,_SS_ and C_min,SS_ of BRI; Figure **(D–F)** for AUC_0-τ,ss_, C_max_,_SS_ and C_min,SS_ of LOR. SC values < 0.1 are not shown. SC values greater than 1.0 are considered sensitive to PK parameters in CSF.

A sensitivity analysis was performed on the model, the results of which are detailed in Figure 5. This analysis demonstrated that LogP was the primary determinant for the CSF AUC_0-τ,ss_, C_max,ss_, and C_min,ss_ of BRI, with all three parameters yielding SC values ≥ 1.0. In contrast, none of the model parameters were sensitive for the corresponding CSF PK parameters of LOR. Additionally, albumin levels and f_up_ notably impacted the CSF PK of both BRI and LOR, while CL_spec_ (P-gp) had a substantial effect on C_max,ss_ of BRI and LOR.

### 3.5 Comparison of the CSF pharmacokinetics between the two ALK inhibitors

**FIGURE 6:**
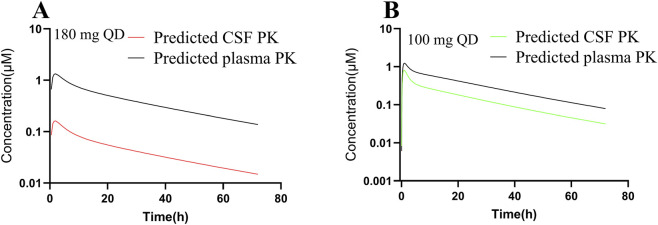
PBPK model-simulated plasma and CSF pharmacokinetics of the two ALK inhibitors. **(A)** Simulated population mean plasma and CSF concentration-time profiles of BRI following a 180 mg QD. **(B)** Simulated population mean plasma and CSF concentration-time profiles of LOR following a 100 mg QD. Solid black lines depict the mean predicted plasma PK profiles, while solid red and green lines indicate the predicted CSF PK profiles for BRI and LOR, respectively.

Although BRI and LOR share similar physicochemical properties, they differ in intrinsic passive transcellular permeability, active efflux clearance, and plasma protein binding. Notably, BRI exhibits significantly higher plasma protein binding than LOR (Supplementary Table S1). Consequently, the CSF PK of BRI differed from that of LOR, characterized by a slower rate and a lower extent of brain penetration (Figure 6). The rate of brain penetration is largely determined by protein binding and passive or active efflux clearance at the BBB (Li et al., 2017). For BRI, higher brain tissue binding coupled with lower passive permeability and higher active efflux clearance accounted for its slower brain penetration. These findings suggest that BBB transport of BRI is co-dominated by protein binding, passive permeability, and efflux clearance, whereas LOR transport is primarily driven by lower efflux clearance.

Additionally, the predicted mean CSF-to-plasma ratio for BRI (K_p,CSF_, 0.20) was close to its brain-to-plasma ratio (K_p,brain_, 0.21); similarly, the mean ratio for LOR (K_p,CSF_, 0.73) closely matched its brain K_p,brain_ (0.86). This indicates that CSF concentrations can reasonably serve as a surrogate for unbound brain concentrations for both BRI and LOR.

### 3.6 Model-informed refinement of therapeutic dosing strategies

**FIGURE 7:**
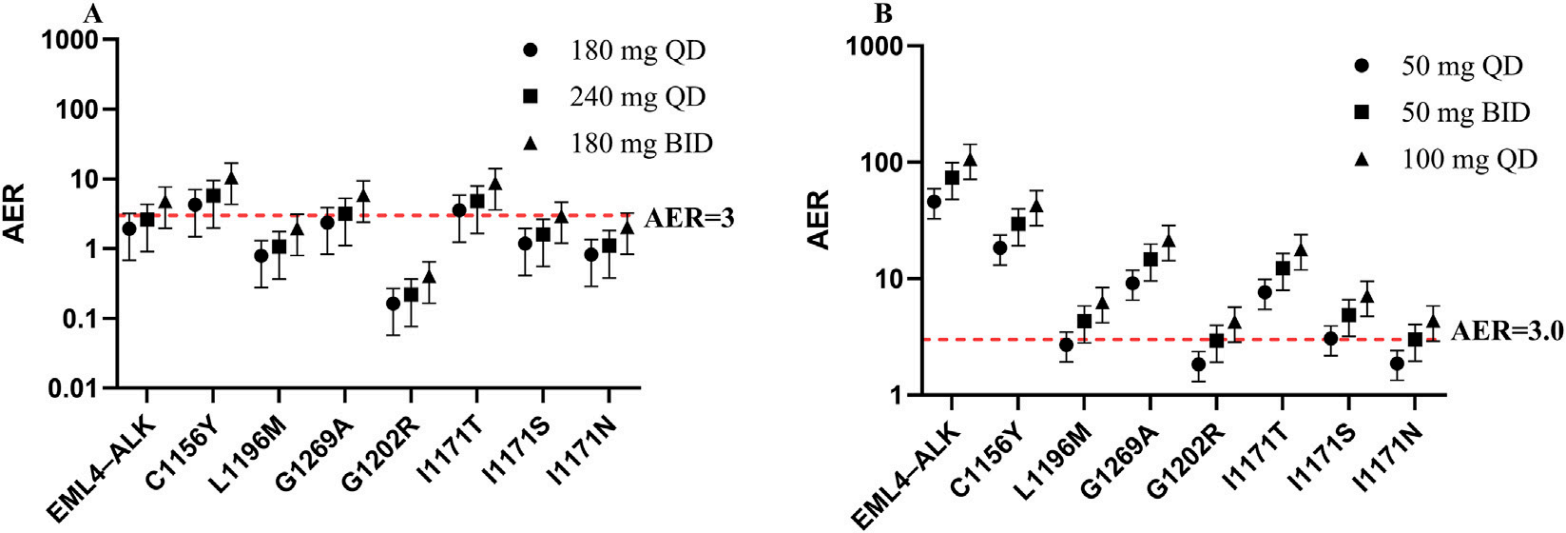
PBPK model-simulated AER values against eight ALK mutations for the two ALK inhibitors in CSF. **(A)** Calculated AER values of BRI following a 180 mg QD,240 mg QD and 180 mg BID. **(B)** Calculated AER values of LOR following a 50 mg QD,50 mg BID and 100 mg QD. The red dot lines indicate a predictor of efficacy for AER = 3.0. Calculated AER values were given with mean ± SD values.

As shown in Figure 7A, the PBPK model-simulated mean AER values for BRI at the standard 180 mg QD regimen all remained below 3.0, with the exception of the value against C1156Y and I1171T. However, for specific ALK mutations (EML4-ALK, C1156Y, G1269A, and I1171T), AER values exceeded the efficacy predictor threshold of AER = 3.0 (indicated by the red dotted line) under the dosing regimens (240 mg QD or 180 mg BID). Although the most of mean AER values for BRI were below 3.0 across the three dosing regimens, they consistently exceeded 1.0 against multiple ALK mutations (Supplementary Table S7), suggesting that BRI may exert a certain therapeutic effect. Figure 7B presents the simulated AER values for LOR under three dosing regimens (50 mg QD, 50 mg BID, and 100 mg QD). With the exception of the mean AER values against mutations L1196M, G1202R, and I1171N at 50 mg QD, which did not reach 3.0, the simulated mean AER values for LOR across the other eight ALK mutations all exceeded the efficacy threshold of 3.0 (red dotted line) under all three regimens. This suggests that LOR has more favorable CNS penetration and potential antitumor activity in the CSF against the tested ALK mutations compared to BRI.

The AER values for both ALK inhibitors exhibited an upward trend with increasing doses, indicating that elevated daily doses may enhance CNS efficacy. A comparison between 50 mg BID and 100 mg QD for LOR reveals differences in AER values attributable to dosing frequency, providing valuable insights for clinical dosing strategy selection and regimen optimization. Similarly, for BRI, the 180 mg BID regimen yielded higher AER values than the 240 mg QD regimen, implying that divided dosing may help maintain more stable drug concentrations in the CSF and thereby optimize CNS therapeutic outcomes. However, it is important to note that the safety and efficacy of the 180 mg BID regimen for BRI have not yet been validated in clinical trials. Furthermore, Supplementary Table S7 summarizes the predicted population mean unbound CSF C_min,ss_, AER values, and the distribution percentage of AER values.

Clinical evidence demonstrates that both BRI and LOR are effective treatments for patients with BM at standard doses (Camidge et al., 2018; Garcia et al., 2024; Solomon et al., 2024; Solomon et al., 2022). The two ALK inhibitors significantly prolonging both time to central nervous system progression and progression-free survival. These clinical trial findings are consistent with and support our model’s predictions.

## 4 Discussion

In this study, we successfully developed and verified a whole-body population PBPK model integrated with a four-compartment brain module for BRI and LOR in cancer patients. The model accurately simulated plasma and CSF pharmacokinetic profiles following repeated dosing. For all evaluated steady-state plasma PK parameters (AUC_0-τ,ss_, C_max,SS_, and C_min,SS_) and CSF PK parameters (C_min,SS_), the predicted-to-observed ratios fell comfortably within the acceptable 0.5- to 2.0-fold range relative to clinical observations (Gettinger et al., 2016; Shaw et al., 2017; Hanley et al., 2023; Chen et al., 2021). Additionally, the predicted K_p,CSF_ values for BRI and LOR also closely matched clinical observations (Shaw et al., 2017; Sato et al., 2024; Sun et al., 2022; de Leeuw et al., 2023). Furthermore, DDI predictions based on CYP3A4 and CYP2C8 kinetics demonstrated excellent consistency with clinical observations (Tugnait et al., 2020; Patel et al., 2020; Chen et al., 2020). These validation results confirm the reliability of the established PBPK model, providing a powerful quantitative tool for evaluating CSF exposure and optimizing dosing regimens for these two ALK inhibitors.

Despite extensive efforts, developing effective therapies for patients with BM remains a significant challenge (Kim et al., 2025). This lack of efficacy often stems from tumor genetic heterogeneity, intrinsic resistance mechanisms, and, critically, the poor penetration of therapeutic agents across the intact human BBB (Pinkiewicz et al., 2025). Successful clinical candidates must cross this barrier to achieve pharmacologically active concentrations. When selecting drugs and designing regimens, key considerations include BBB permeability, CSF PK, and target inhibition potency (Pinkiewicz et al., 2025; Cornelissen et al., 2023). The K_p,CSF_ value, which measures the extent of brain penetration, is a fundamental parameter in drug development for patients with BM (Zou et al., 2025). However, pharmacologically active brain exposure is dictated by K_p,CSF_, systemic plasma exposure, and f_up_. Our PBPK simulations, supported by *in vitro* permeability and transporter kinetics, indicate that LOR transport across the BBB is dominated by passive permeability (K_p,CSF_≈0.8), whereas BRI transport (K_p,CSF_ = 0.20) is primarily restricted by P-gp-mediated efflux. Consequently, while LOR’s brain penetration is approximately 4-fold greater than BRI’s, its C_min,SS_ is roughly 10-fold higher under standard dosing (Supplementary Table S7). This elevated CSF exposure is largely driven by LOR’s superior unbound plasma concentrations. Thus, the combination of a higher K_p,CSF_ and higher fraction unbound in plasma enables LOR to adequately inhibit ALK variants in the brain at standard doses.

It should be noted that obtaining human CSF samples is clinically challenging, leading to extremely limited clinical data available for PBPK model validation, which is a common challenge in this field of research. In this study, clinical data from three studies were used to verify the PBPK model-predicted LOR concentrations in CSF, whereas only one clinical study reporting BRI concentrations in CSF was identified. A clinical review documented the exposure ratio of free BRI in CSF to plasma (0.31) (Gil et al., 2023), which aligns closely with our model predictions. Furthermore, to verify the accuracy of central BRI concentration predictions, we incorporated two animal studies assessing BRI distribution in rat and mouse brain tissues. The predicted brain-to-plasma exposure ratios were consistent with the experimental values (Sparidans et al., 2018; Li et al., 2019). While the sparse dataset limits the absolute precision of simulated CSF concentrations, the model’s reliability is bolstered by cross-species verification and mechanistic modeling. The model accurately replicated rodent brain distribution, validating the physiological parameters of BBB permeability. Furthermore, as predictions are based on physicochemical properties rather than empirical fitting to a single clinical point, the mechanistic framework provides reasonable confidence in the predicted BRI CNS exposure despite limited direct clinical validation. Overall, despite the limited clinical CSF data for BRI, the model’s performance is supported by the available human and animal evidence.

Hypoproteinemia is a highly prevalent complication in oncology patients (Teigell Muñoz et al., 2026), serving not only as an indicator of systemic inflammation and malnutrition but also as a critical modifier of PK profiles (Alsuhebany et al., 2026). As the primary binding protein in plasma, a significant decline in albumin concentration directly elevates the f_up_. This amplification effect is particularly pronounced for highly protein-bound antineoplastic agents (Celestin and Musteata, 2021). Furthermore, our model sensitivity analysis confirmed that plasma albumin levels substantially influence CSF PK parameters (Figure 5). This mechanistic link underscores the rationale for adjusting plasma albumin levels in our cancer population model. Interestingly, LOR exhibited minimal sensitivity to most model parameters compared to BRI. This lack of sensitivity is likely mechanistically driven by LOR’s high passive permeability across the BBB, which renders its brain distribution less dependent on active efflux transporters such as P-gp. Furthermore, LOR’s lower plasma protein binding relative to BRI insulates its free fraction from fluctuations in albumin levels. These properties contribute to a more consistent and predictable CSF exposure profile for LOR, reducing the impact of physiological variability on its PK in CSF.

Target inhibition potency represents another critical determinant of clinical efficacy. Given that adequate target suppression is a prerequisite for success, the AER-which integrates unbound CSF concentrations with inhibitory potency-serves as an optimal criterion for selecting BM therapies. We established an AER ≥3.0 in the CSF as the threshold for effectively inhibiting various ALK variants based on the published paper (Sun et al., 2022), particularly within microscopic tumors shielded by an intact BBB. While both agents are potent against multiple ALK variants, LOR’s excellent BBB penetration (K_p,CSF_∼0.80), reasonably high f_up_, and high overall exposure (100 mg QD) yield an AER≥3.0 for all eight evaluated variants at standard doses. Consistent with our model, clinical evidence confirms that while both BRI and LOR are effective against BM at standard doses (Camidge et al., 2018; Garcia et al., 2024; Solomon et al., 2024; Solomon et al., 2022). Conversely, due to lower brain exposure and modest potency against specific mutations (L1196M, G1202R, I1171S, and I1171N), BRI fails to achieve an AER≥ 3.0 for these variants even at an escalated dose of 240 mg QD or 180 mg BID. Additionally, among patients previously treated with second-generation ALK inhibitors (including BRI), G1202R represents the most prevalent acquired resistance mutation, with a detection rate of 53% in circulating cell-free DNA and 55% in tumor tissue (Shaw et al., 2019). Despite this, LOR retained substantial activity against G1202R-positive disease, achieving an objective response rate of 57% and a median progression-free survival of 8.2 months (Gruber et al., 2024). This clinical selectivity profile aligns with the directional output of our PBPK model, which predicted a markedly superior mean AER for LOR (4.3 at the standard dose of 100 mg QD) compared with BRI (0.16 at the standard dose of 180 mg QD). Collectively, these simulations identify LOR as the optimal ALK inhibitor across the evaluated variants. For two specific variants (C1156Y and I1171T), BRI remains a viable option at standard doses.

While recent studies have demonstrated the utility of PBPK models in predicting brain pharmacokinetic behavior, and emerging hybrid approaches integrating deep learning with mechanistic modeling are accelerating these predictions (Zou et al., 2025; Gruber et al., 2024), there remains a critical need for models that accurately account for the interplay between passive permeability and active efflux transport. Our findings align with recent observations indicating that successful CNS penetration requires a delicate balance between high K_p,brain_ and a low efflux ratio (Li et al., 2021). Similar to recent PBPK evaluations of CDK4/6 inhibitors for BM (Zhan et al., 2025), we emphasize the following novel aspects of our study: (1) disease-specific application: unlike generic PBPK models often based on healthy physiology, our model incorporates specific pathophysiological alterations in NSCLC patients (e.g., hypoalbuminemia and delayed gastric emptying). This integration provides a more realistic and predictive framework for this vulnerable patient population. (2) quantitative efficacy threshold (AER): We introduce the AER≥3.0 as a mechanistic bridge linking CSF PK to pharmacodynamic outcomes. This approach offers a practical tool for dose optimization in patients with BM, addressing a significant gap in existing literature that focuses predominantly on PK-only endpoints.

Several limitations should be acknowledged when interpreting these PBPK results. First, clinical CSF data for BRI are restricted to trough concentrations from a single clinical trial, lacking broader external validation in larger cohorts. Second, the simulated efficacy of reduced LOR dose (50 mg QD or BID) remains theoretical, pending clinical verification. Finally, P-gp, as an important efflux transporter on the endothelial cells of the BBB and blood-tumor barrier, is often significantly upregulated by the tumor microenvironment (Rijmers et al., 2025). Therefore, this potential P-gp overexpression within the brain tumor microenvironment may introduce additional uncertainty regarding BRI’s predicted brain exposure. Overall, dosing refinement for BRI and LOR still requires clinical observation and verification. Although PBPK simulation results cannot predict final clinical outcomes, they can still provide valuable guidance for the medication of NSCLC patients with BM.

## 5 Conclusion

In conclusion, a whole-body population PBPK model integrated with a four-compartment brain module was successfully developed and verified to simulate the plasma and CSF PK of BRI and LOR in cancer patients. By introducing the AER, which integrates unbound CSF exposure with target inhibitory potency, this study established a quantitative threshold (AER≥3.0) for informing dosing strategies for patients with BM. Model simulations demonstrated that LOR (100 mg QD) achieved an AER≥3.0 against all eight evaluated ALK variants. In contrast, even at doses of 180 mg BID or 240 mg QD, BRI only provided sufficient target coverage for four specific variants: EML4-ALK, C1156Y, G1269A, and I1171T. Overall, this study provides a mechanistic pharmacokinetic framework highlighting that K_p,CSF_, f_up_, and target inhibitory potency are critical for CNS drug discovery.

## Data Availability

The original contributions presented in the study are included in the article/Supplementary Material, further inquiries can be directed to the corresponding author.
