# Supplementary material for: Physiologically based pharmacokinetic model of two ALK inhibitors to inform drug and dosing regimens for brain metastasis patients
Source: Front Pharmacol. 2026 Jul 30;17:1901617. doi: 10.3389/fphar.2026.1901617 (PMC13468919; doi:10.3389/fphar.2026.1901617)
Supplement: Supplementary file 1 [file Supplementaryfile1.docx]

**Table S1**	Drug-dependent parameters of the final BRI and LOR PBPK model.

| Parameter (Units) | | BRI | LOR ^[18,22]^ | Descriptions and comments |
| --- | --- | --- | --- | --- |
| MW (g/mol) | | 584.1 | 406.4 | Molecular weight |
| pKa (Basic) | | 3.65, 4.72,8.07^[23]^ | 4.9 | Base dissociation constant |
| Log P | | 3.02^[23]^ | 2.47 | Lipophilicity |
| Solubility (mg/mL) | | 0.10 ^[24]^ | 0.11 | - |
| Specific intestinal permeability(cm/s) | | P_app_:2.57🞨10^-6 [25]^ | P_app_: 28🞨10^-6^ | - |
| f_up_ | | 0.09 ^[26]^ | 0.34 | Free fraction in plasma |
| R_bp_ | | 1.1(Calculated) | 1.1(Calculated) | Blood-to-plasma concentration ratio |
| CL_R_ (L/h) | | GFR*f_up_ |  | Renal clearance |
| GFR fraction | | 1.0 (Default) | | Fraction of filtered drug in the urine |
| Weibull time (min) | | 45 (Optimized） | 30 (Optimized） | Dissolution time of 50% drug |
| Weibull shape | | 0.92 (Default） | | Shape parameter of Weibull function |
| Cell permeabilities | | PK-Sim Standard | | - |
| Partition coefficients | | Rodgers and Rowland | | - |
|  | CL_spec_(CYP3A4) (μL/min/mg) | 9.41 protein ^[25]^ | 0.0018^a^,0.0226^b^ (pmol/min/pmol) | Specific clearance for CYP3A4. LOR converting to M2a (a) and M6(b) |
| Metabolic parameters | CL_spec_(CYP2C8)(μL/min/mg) | 2.79 protein ^[25]^ | 0.00036 (pmol/min/pmol) | Specific clearance for CYP2C8, LOR converting to M2a |
|  | CL_spec_(CYP2C19) (μL/min/mg) | - | 0.0089(pmol/min/pmol) | Specific clearance for CYP2C19, LOR converting to M2a |
|  | CL_spec_(P-gp) (nmol/min/g tissue) | 0.10^[27]^ | 0.04 | Specific efflux for P-gp |
|  | CL_spec_(CYP3A5) (μL/min/mg) | - | 0.011(pmol/min/pmol) | Specific clearance for CYP3A5, LOR converting to M2a |
|  | UGT1A3(pmol/min/pmol) | - | 0.00078 | Specific clearance for UGT1A3, LOR converting to M1a |
|  | UGT1A4(pmol/min/pmol) | - | 0.0023 | Specific clearance for UGT1A4, LOR converting to M1a |
| Concentration (μM/L liver tissue) | CYP3A4 | 3.02^[28]^ | | Reference concentration set in PK-Sim for CYP3A4, CYP3A5, CYP2C8, CYP2C19, UGT1A3, UGT1A4, and P-gp |
|  | CYP3A5 | 0.04^[18]^ | |  |
|  | CYP2C8 | 2.56^[28]^ | |  |
|  | CYP2C19 | 0.51^[18]^ | |  |
|  | UGT1A3 | 0.53^[18]^ | |  |
|  | UGT1A4 | 0.25^[18]^ | |  |
|  | P-gp | 1.30^[20]^ | |  |
| K_i_ P-gp(μM) | | 1.65^[25]^ | 2.99 |  |
| EC_50_ CYP3A4 (μM) | |  | 0.29 |  |
| E_max_ CYP3A4 | |  | 5.99 |  |
| GET(min) | | 190^[28]^ | | gastric emptying time |
| Hematocrit | | 0.33^[28]^ | | - |
| Albumin level (g/dl) | | 3.1^[28]^ | | - |

**Table S2** Fractional contribution of metabolic enzymes

| Inhibitors | Metabolites | rhCYP Isoform | Cl_int_(μL/minl/mg protein) | ISEF | Abundance | scaled velocity | Contribution (%) |
| --- | --- | --- | --- | --- | --- | --- | --- |
| BRI | M36 | CYP3A4 | 5.24 | 0.33 | 137 | 3.526 | 79.1 |
|  |  | CYP2C8 | 2.59 | 1.68 | 24 | 0.933 | 20.9 |
|  | Others | CYP3A4 | 4.17 | 0.33 | 137 | - | - |
|  |  | CYP2C8 | 0.20 | 1.68 | 24 | - | - |
| Inhibitors | Metabolites | rhCYP Isoform | Cl_int_(pmol/minl/pmol) | ISEF | Abundance | scaled velocity | Contribution (%) |
| LOR | M6 | CYP3A4 | 0.0226 | 0.33 | 137 | 1.0217 | 100.0 |
|  | M2a | CYP3A4 | 0.0018 | 0.33 | 137 | 0.0814 | 33.3 |
|  |  | CYP2C8 | 0.00036 | 1.68 | 24 | 0.0145 | 5.9 |
|  |  | CYP3A5 | 0.011 | 0.12 | 103 | 0.1360 | 55.6 |
|  |  | CYP2C19 | 0.0036 | 0.25 | 14 | 0.0126 | 5.2 |
|  | M1a | UGT1A3 | 0.00078 | 1 | 15.3 | 0.0119 | 10.5 |
|  |  | UGT1A4 | 0.0023 | 1 | 44.3 | 0.1019 | 89.5 |

**Table S3** BRI and LOR activity against various ALK mutations

| ALK mutations | ALK mean IC_50_ (nM) | |
| --- | --- | --- |
|  | BRI | LOR |
| EML4–ALK | 11 | 2 |
| C1156Y | 5 | 5 |
| L1196M | 27 | 34 |
| G1269A | 9 | 10 |
| G1202R | 130 | 50 |
| I1171T | 6 | 12 |
| I1171S | 18 | 30 |
| I1171N | 26 | 49 |

**Table S4** Comparison of mean plasma pharmacokinetic parameters between predicted and observed values in patients with ALK-positive NSCLC

| Clinical studies | Inhibitors | Dosing regimens | AUC_0-τ,ss_ (ng·h/mL, SD) | | C_max,SS_ (ng/mL, SD) | | C_min,SS_ (ng/mL, SD) | | Prediction/observation ratio | | |
| --- | --- | --- | --- | --- | --- | --- | --- | --- | --- | --- | --- |
|  |  |  | Prediction | Observation | Prediction | Observation | Prediction | Observation | AUC_0-τ,ss_ | C_max,SS_ | C_min,SS_ |
| Gettinger et al | BRI^a^ | 30 mg QD | 3546.6 (1523.3) | 2689 | 228.8 (71.8) | 249.5 | 98.8 (56.7) | 77.5 | 1.32 | 0.92 | 1.27 |
|  |  | 60 mg QD | 7024.9 | 5069 (1665) | 454.5 (141.5) | 491.6 (223.9) | 194.8 (111.3) | 141.4 (50.5) | 1.39 | 0.92 | 1.38 |
|  |  | 90 mg QD | 10672 (4607.9) | 9142 (4077) | 687.9 (216.6) | 634.1 (310.1) | 297.6 (171.3) | 255.2 (127.4) | 1.17 | 1.08 | 1.17 |
|  |  | 120 mg QD | 14098 (6050) | 13888 (7011) | 911.2(285.0) | 942.3 (472.3) | 391.5 (224.4) | 353.7 (189.6) | 1.02 | 0.97 | 1.11 |
|  |  | 180 mg QD | 21213 (9138) | 23478 (14463) | 1370.0 (430.2) | 1694.3 (1014.3) | 589.7 (339.2) | 615.0 (408.2) | 0.90 | 0.81 | 0.96 |
|  |  | 240 mg QD | 28363 (12258) | 30117 (19921) | 1830.4 (576.7) | 2280.0 (1308.5) | 789.3 (455.4) | 725.2 (418.3) | 0.94 | 0.80 | 1.09 |
| Hanley et al |  | 180 mg QD | - | - | - | - | 619.1 (403.1) | 589.0 | - | - | 1.05 |
| Shaw et al | LOR^b^ | 10 mg QD | 825 (480.5) | 820 | 74.0 (60.7) | 74 | 18.8 (7.2) | - | 1.01 | 1.00 | - |
|  |  | 25 mg QD | 1793 (1115) | 1708 (29) | 147.3 (76.3) | 138 (35) | 35.8 (21.8) | - | 1.05 | 1.07 | - |
|  |  | 50 mg QD | 3269 (2272) | 3487 (41) | 301.3 (209.6) | 360 (27) | 59.3 (25.0) | - | 0.94 | 0.84 | - |
|  |  | 75 mg QD | 4721 (3575) | 4117 (55) | 447.8 (237.3) | 422 (50) | 81.7 (38.3) | - | 1.15 | 1.06 | - |
|  |  | 100 mg QD | 5394 (5021) | 5065 (32) | 595.4 (319) | 569 (32) | 101.3 (54.4) | - | 1.06 | 1.05 | - |
|  |  | 150 mg QD | 7910 (8316) | 6185 | 894.6 (640.1) | 638 | 145.6 (97.6) | - | 1.28 | 1.40 | - |
|  |  | 200 mg QD | 10533 | 7856 | 1200 (645.4) | 1042 | 194.0 (157.4) | - | 1.34 | 1.15 | - |
| Chen et al |  | 100 mg QD | 7165 (1485) | 9088 (35) | 667.2 (470.7) | 695 (40) | 149.2 (30.1) | 133 | 0.79 | 0.96 | 1.12 |

^a^: arithmetic mean values are shown; ^b^: geometric mean values are shown; SD: standard deviation; -: not reported data.

**Table S5** Brain distribution studies of BRI in animals

| Studies | Animals | Dosage | n | Male/ Female | Brain-to-plasma exposure ratio | |
| --- | --- | --- | --- | --- | --- | --- |
|  |  |  |  |  | Observation | Prediction |
| Li et al | SD Rat | 5 mg/kg | 60 | Male | 0.22^a^ | 0.20 |
| Sparidans et al | FVB/NRj mouse | 25 mg/kg | 6 | Male | 0.12^b^ | 0.17 |

^a^：0-6 h, ^b^:0-24h.

**Table S6** PK parameters changes of BRI and LOR in DDIs

| Victim | Perpetrators | Dosing regimens | Parameters | Predicted ratio | Observed ratio | Predicted/observed |
| --- | --- | --- | --- | --- | --- | --- |
| BRI | GEM | BRI 90 mg QD+GEM 600 mg BID | AUC_0-inf_ (ng·h·mL^-1^) | 1.29 | 0.88 | 1.47 |
|  |  |  | C_max_ (ng/mL) | 1.02 | 0.59 | 1.73 |
|  | ITR | BRI 90 mg QD+ITR 200 mg BID | AUC_0-inf_ (ng·h·mL^-1^) | 1.82 | 2.01 | 0.91 |
|  |  |  | C_max_ (ng/mL) | 1.11 | 1.21 | 0.92 |
|  | RIF | BRI 180 mg QD+RIT 600 mg QD | AUC_0-inf_ (ng·h·mL^-1^) | 0.21 | 0.20 | 1.05 |
|  |  |  | C_max_ (ng/mL) | 0.62 | 0.40 | 1.55 |
| LOR | ITR | LOR 100 mg QD+ITR 200 mg QD | AUC_0-inf_ (ng·h·mL^-1^) | 1.44 | 1.41 | 0.99 |
|  |  |  | C_max_ (ng/mL) | 1.39 | 1.24 | 1.16 |
|  | RIF | LOR 100 mg QD+ RIT 600 mg QD | AUC_0-inf_ (ng·h·mL^-1^) | 0.08 | 0.15 | 0.53 |
|  |  |  | C_max_ (ng/mL) | 0.48 | 0.24 | 2.0 |

**Table S7** PBPK model-predicted unbound BRI and LOR exposure and AER in the CSF at the steady-state following the standard or modified dosing regimens

| CSF | Inhibitors | AER values | AER value distribution | AER values | AER value distribution | AER values | AER value distribution |
| --- | --- | --- | --- | --- | --- | --- | --- |
|  |  | Standard (180 mg QD,14 days) | AER≥1(%)/AER≥3(%) | Alternative (240 mg QD,14 days) | AER≥1(%)/AER≥3(%) | Alternative (180 mg BID,14 days) | AER≥1(%)/AER≥3(%) |
| Unbound C_min,SS_ (nM, mean±SD) | BRI | 21.3±13.8 | - | 28.7±18 | - | 53.0±31.4 | - |
| EML4–ALK (mean±SD) |  | 1.9±1.3 | 71/21 | 2.6±1.7 | 79/33 | 4.8±2.9 | 100/67 |
| C1156Y(mean±SD) |  | 4.3±2.8 | 96/58 | 5.7±3.8 | 100/67 | 10.6±6.3 | 100/96 |
| L1196M(mean±SD) |  | 0.8±0.5 | 33/0 | 1.1±0.7 | 46/0 | 2.0±1.2 | 75/21 |
| G1269A (mean±SD) |  | 2.4±1.5 | 79/3 | 3.2±2.1 | 92/46 | 5.9±3.5 | 100/75 |
| G1202R (mean±SD) |  | 0.2±0.1 | 0/0 | 0.2±0.1 | 0/0 | 0.4±0.2 | 0/0 |
| I1171T (mean±SD) |  | 3.6±2.3 | 92/50 | 4.8±3.1 | 100/58 | 8.8±5.2 | 100/92 |
| I1171S(mean±SD) |  | 1.2±0.8 | 50/0 | 1.6±1.0 | 58/17 | 2.9±1.7 | 92/42 |
| I1171N (mean±SD) |  | 0.8±0.5 | 33/0 | 1.1±0.7 | 46/0 | 2.0±1.2 | 75/25 |
|  | LOR | Standard (100 mg QD,14 days) | AER≥3(%) | Alternative (50 mg QD,14 days) | AER≥3(%) | Alternative (50 mg BID,14 days) | AER≥3(%) |
| C_min,SS_ (nM, mean±SD) |  | 214.1±71.6 | - | 91.7±26.3 | - | 146.9±51.1 | - |
| EML4–ALK (mean±SD) |  | 107.0±35.8 | 100 | 45.9±13.2 | 100 | 73.4±25.5 | 100 |
| C1156Y(mean±SD) |  | 42.8±14.3 | 100 | 18.3±5.3 | 100 | 29.4±10.2 | 100 |
| L1196M(mean±SD) |  | 6.3±2.1 | 100 | 2.7±0.8 | 30 | 4.3±1.5 | 80 |
| G1269A (mean±SD) |  | 21.4±7.2 | 100 | 9.2±2.6 | 100 | 14.7±5.1 | 100 |
| G1202R (mean±SD) |  | 4.3±1.4 | 70 | 1.8±0.5 | 0 | 2.9±1.0 | 30 |
| I1171T (mean±SD) |  | 17.8±6.0 | 100 | 7.6±2.2 | 100 | 12.2±4.3 | 100 |
| I1171S(mean±SD) |  | 7.1±2.4 | 100 | 3.1±0.9 | 60 | 4.9±1.7 | 100 |
| I1171N (mean±SD) |  | 4.4±1.5 | 70 | 1.9±0.5 | 0 | 3.0±1.0 | 30 |

C_ss,min_: trough steady-state concentration; Ranges are expressed as minimum and maximum values; AER: ALK engagement ratio; SD：standard deviation; QD: once daily; BID: twice daily.
